# BiSpec Pairwise AI: guiding the selection of bispecific antibody target combinations with pairwise learning and GPT augmentation

**DOI:** 10.1007/s00432-024-05740-3

**Published:** 2024-05-07

**Authors:** Xin Zhang, Huiyu Wang, Chunyun Sun

**Affiliations:** 1 Beijing Engineering Research Center of Protein and Antibody, Sinocelltech Ltd., Beijing, 100176 China; 2 School of Medicine, Nankai University, Tianjin, 300071 China

**Keywords:** Bispecific antibody, Target combination, Machine learning, Pairwise learning, GPT

## Abstract

**Purpose:**

Bispecific antibodies (BsAbs), capable of targeting two antigens simultaneously, represent a significant advancement by employing dual mechanisms of action for tumor suppression. However, how to pair targets to develop effective and safe bispecific drugs is a major challenge for pharmaceutical companies.

**Methods:**

Using machine learning models, we refined the biological characteristics of currently approved or in clinical development BsAbs and analyzed hundreds of membrane proteins as bispecific targets to predict the likelihood of successful drug development for various target combinations. Moreover, to enhance the interpretability of prediction results in bispecific target combination, we combined machine learning models with Large Language Models (LLMs). Through a Retrieval-Augmented Generation (RAG) approach, we supplement each pair of bispecific targets’ machine learning prediction with important features and rationales, generating interpretable analytical reports.

**Results:**

In this study, the XGBoost model with pairwise learning was employed to predict the druggability of BsAbs. By analyzing extensive data on BsAbs and designing features from perspectives such as target activity, safety, cell type specificity, pathway mechanism, and gene embedding representation, our model is able to predict target combinations of BsAbs with high market potential. Specifically, we integrated XGBoost with the GPT model to discuss the efficacy of each bispecific target pair, thereby aiding the decision-making for drug developers.

**Conclusion:**

The novelty of this study lies in the integration of machine learning and GPT techniques to provide a novel framework for the design of BsAbs drugs. This holistic approach not only improves prediction accuracy, but also enhances the interpretability and innovativeness of drug design.

**Supplementary Information:**

The online version contains supplementary material available at 10.1007/s00432-024-05740-3.

## Introduction

Malignant tumors represent a major global health challenge, often culminating in mortality due to the limitations of conventional treatments like surgery, radiation therapy and chemotherapy (Obradovic 2023). Fortunately, immunotherapy represented by immune checkpoints such as PD-(L)1, has emerged as a revolutionary approach that aims to inhibit immune checkpoints (Ren et al. 2020), thereby activating the immune system and enhancing its capacity to recognize and eradicate tumor cells (Teige et al. 2019). In recent years, monoclonal antibody (mAb) therapies have been widely adopted in cancer treatment, with combination therapies further enhancing efficacy and maintaining manageable safety profiles. However, the long-term efficacy of mAbs is limited by complex resistance mechanisms in the tumor microenvironment. For instance, in patients treated with PD-1 antibody, other immune checkpoints are significantly up-expressed and cause T exhaustion, leading to drug resistance (Zhou et al. 2023a, b). As BsAbs can directly target immune cells to tumors, or simultaneously target multiple tolerance pathways, drug resistance and severe adverse reactions are greatly reduced. Besides, BsAbs offer greater clinical administration convenience and potential synergistic effects superior to mAb combination therapies. BsAbs have triggered a current surge in next generation anti-tumor drugs.

Several BsAbs have successfully been approved, sharing common characteristics such as target specificity to cell types (tumor cells, endothelial cells, and immune-tolerant CD8^+^T cells) prevalent within the tumor microenvironment while exhibiting lower expression in normal tissues, thereby ensuring a higher safety margin. These BsAbs therapies encompass various strategies, including: (1) tumor-associated antigen (TAA) coupled to T cell activators (e.g., glofitamab (Shirley 2023) targeting CD20 and CD3, relying on the high expression of CD20 on tumor cells to cross-link antibodies into clusters, enhancing the activity of CD3, thereby improving the ability of T cells to kill tumor cells); (2) dual immune checkpoint inhibitors (ICI) (e.g., AK104 (Keam 2022) targeting PD-1 and CTLA4 expressed singly/doubly on T cells, dual blockade of immune tolerance, restoring T cell exhaustion); (3) combinations of immune checkpoint inhibitors with anti-angiogenesis antibodies (e.g., AK112 (Zhao et al. 2023) targeting PD-1 and VEGF, inhibiting tumors through blocking immune tolerance and anti-angiogenesis mechanisms); (4) dual-targeting TAA (e.g., amivantamab (Chon et al. 2023; Syed 2021; Vyse and Huang 2022; C. Zhou et al. 2023a, b), a BsAbs targeting EGFR and c-MET, was approved in 2021 for the treatment of Non-small cell lung cancer (NSCLC), bridging two antigens expressed singly/doubly on tumor cells, simultaneously blocking two tumor growth signaling pathways).

The development of BsAbs still faces many challenges, especially how to identify target pairs to achieve synergistic effects, while considering whether there are optimal strategies to minimize the toxicity of BsAbs (Thakur et al. 2018). The current focus in the development of BsAbs is skewed towards leveraging established monoclonal antibody targets, either already approved or in clinical trials. However, how to predict the success rate of target combinations for BsAbs, thereby reducing the cost of trials, may be a current challenge in the pharmaceutical industry. Besides, how to combine different targets within a bispecific drug to achieve the best efficacy and safety will be the key issue that researchers need to consider.

This endeavor necessitates a deep understanding of the spatial and temporal expression characteristics of targets within the tumor microenvironment, a task for which single-cell RNA sequencing (scRNA-seq) offers unprecedented insights into gene expression profiles in the tumor microenvironment at the level of individual cells (Vallejos et al. 2016; Van de Sande et al. 2023). Despite the growing body of research on BsAbs in tumor immunotherapy, a direct integration of scRNA-seq data with bispecific drug design remains largely unexplored.

The drug discovery and development process is remains lengthy, expensive, and with a high failure rate. Fortunately, the advent of artificial intelligence technologies, coupled with the utilization of large datasets generated by various high-throughput techniques, is streamlining research and development efforts, thereby expediting the delivery of new drugs to patients (Liu et al. 2021). Computational techniques such as machine learning and deep learning have been widely applied in drug development processes. Innovations include machine learning frameworks like iBCe-eL that combines extremely randomized trees (ERT) and gradient boosting (GB) classifiers (Manavalan et al. 2018) for the prediction of B cell epitope, and novel approaches such as using Bayesian models for high-affinity single-chain variable fragments (scFvs) library design (Li et al. 2023). Tools like ABlooper (Abanades et al. 2022) for the structure prediction of antibody complementarity determining regions (CDRs) loop, machine learning classifiers for designing antibody humanization to reduce immunogenicity (Marks et al. 2021), Parapred (Liberis et al. 2018) employing convolutional and recurrent neural networks to predict antibody–antigen binding sites, and mmCSM-AB (Myung et al. 2020) for assessing the impact of multiple mutations in antibodies on antigen binding affinity illustrate the diverse applications of AI in enhancing targeting accuracy and efficacy in drug development. Despite limitations such as sample size and the scarcity of large datasets, computational designs have not been widely utilized in the development of BsAbs. Nonetheless, several researchers have begun to apply computational models, including machine learning, to BsAbs development, making significant strides. Baker et al. (2019) reported on the development of an improved nonreduced peptide map method coupled with machine learning to enable rapid identification of disulfide bonds and cysteine-related variants in IgG1 knob-into-hole BsAbs. Multi-scale model calculations have also been used to simulate the spatio-temporal dynamics of three-body interactions among BsAb, CD3 and TAA to maximize drug efficacy and avoid off-target effects (Su et al. 2024). In addition, computational and rational engineering were used to design heavy chain/κ light chain interface for expressing fully BsAbs (Froning et al. 2017). The use of molecular modeling has improved the stability of the T receptors, allowing pairing with antibodies to form bispecifics (Froning et al. 2020). However, these studies have not focused on the target selection for bispecific antibody therapeutics.

In the past, integration of machine learning with drug design, it was usually only possible to provide the final prediction results, but there was a lack of interpretable explanations. This makes it difficult for drug designers to decide whether they should refer to the results of the machine learning models. Generative Pretrained Transformers (GPT), particularly the acclaimed ChatGPT, are at the forefront of an AI revolution. GPT can leverage its prior pre-trained knowledge and the latest research advancements obtained through search augmentation, combined with our task-specific custom prompts, to generate reports in natural language with enhanced interpretability. Chen et al. (2023) evaluated the capabilities of ChatGPT in processing and understanding biomedical corpora. Yang et al. (2022) developed a pre-trained deep neural network-based model (scBERT) for cell annotation in scRNA-seq data. These studies discuss the positive impact of GPT on bioinformatics.

In this study, we proposed a BSPAI (BiSpec Pairwise AI) framework to accurately predict the optimal target pairs in developing BsAbs. Specifically, our model used gene expression data from different cell types in single-cell transcriptomics and a list of BsAbs currently approved and in clinical trials as inputs. It employed a rich set of biological features such as the double positive proportion of target pairs, safety, mechanism, gene embeddings, etc., with each target pair as the output to predict the likelihood of that pair being successfully approved as a bispecific drug. We compared different machine learning models and conducted a detailed analysis of each type of biological feature. Ultimately, combining the XGBoost model with pairwise learning achieved the best AUC (area under the ROC curve) of 89.29%. Additionally, we integrated GPT to enhance interpretability. We believe that our approach is an effective tool for providing candidates for new target combinations, for further validation in wet lab experiments. The contributions of our work are mainly in the following three aspects:

1. Based on biological knowledge and experience, we provided a variety of biological features that effectively improved the accuracy of bispecific drug design prediction;
2. We compared different machine learning models, combining the XGBoost model with pairwise learning, effectively mitigating the issue of insufficient positive samples in clinical trials, achieving the best AUC of 89.29%;
3. GPT was utilized to enhance the interpretability of each bispecific target pair, thereby aiding decision-making for drug developers.

## Methods

### Task definition

**Fig. 1:**
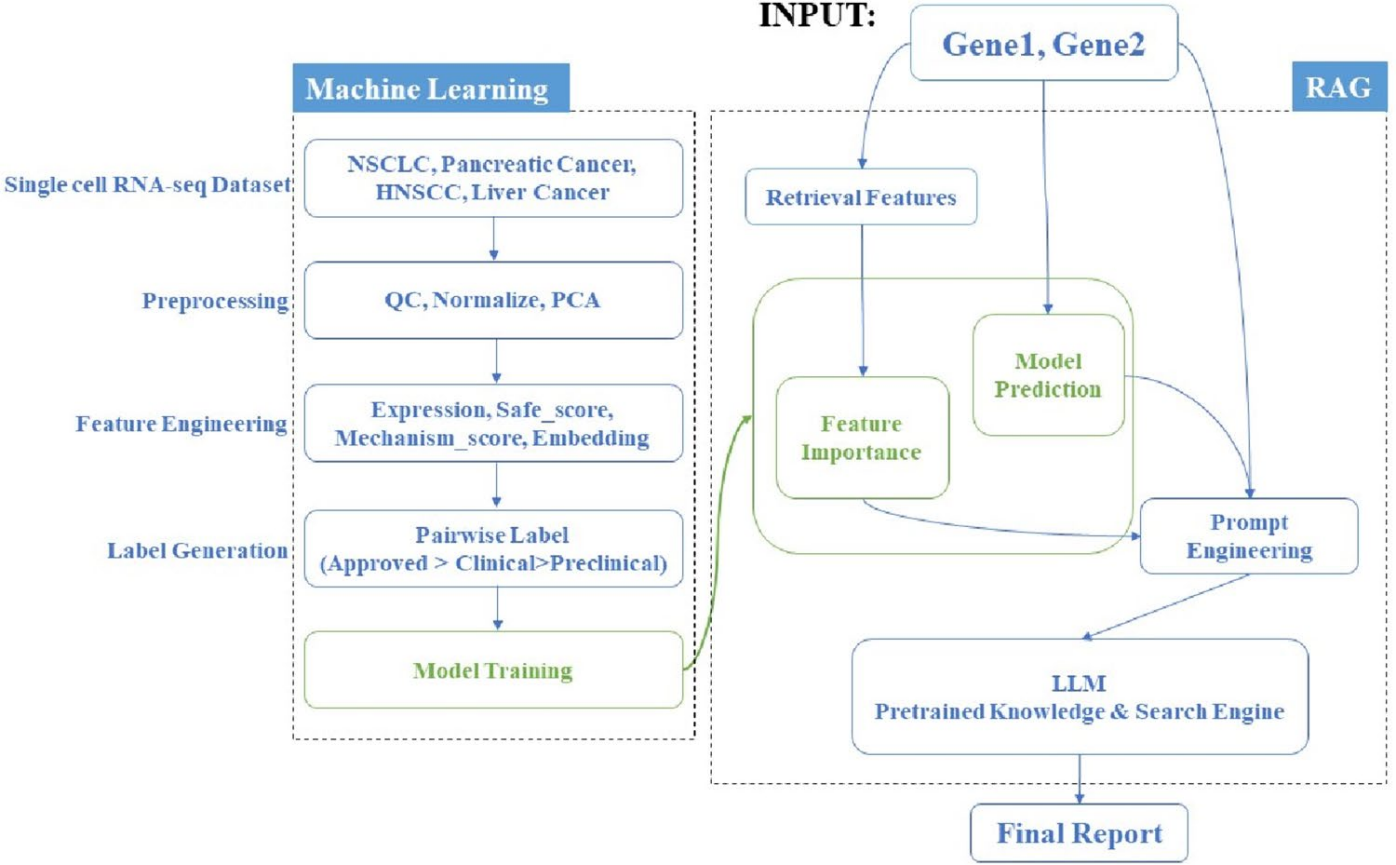
BSPAI consists of two phases: Phase 1, feature extraction and model training, uses a dataset of bispecific targets to predict target synergy and identify key features influencing these predictions. In Phase 2, enhanced interpretation through LLM integration, the model synthesizes initial findings into a prompt for an LLM, which generates an interpretive report. This approach combines predictive analytics with contextual intelligence, offering a comprehensive understanding of bispecific target combinations’ potential

In this segment of the article, we delineate the methodology employed in constructing the BSPAI model, a sophisticated computational framework designed to refine the selection process for bispecific target combinations. Illustrated in Fig. 1, the BSPAI model’s primary objective is to adeptly forecast the most promising target pair combinations from a given set of inputs, culminating in the generation of an interpretive report that is both precise and comprehensive.

The BSPAI model operates through a bifurcated approach:

#### Phase 1: Feature extraction and model training

Initially, the model is calibrated using a dataset comprised of BsAbs which are either already approved or undergoing clinical progress. This phase involves a meticulous extraction of features across multiple dimensions, facilitating a nuanced analysis of potential target combinations. The machine learning algorithm employed in this phase is tailored to predict the synergy of two given targets, outputting a probabilistic value indicative of their combined efficacy. Concurrently, it identifies and highlights the pivotal features influencing the prediction, thereby ensuring transparency and insight into the decision-making process.

#### Phase 2: Enhanced interpretation through LLM integration

Subsequently, the model enters an enhancement phase where the predictions and key features identified in the initial phase are synthesized into a comprehensive prompt. This prompt is then inputted into an LLM, which leverages its extensive pre-trained knowledge and search engine insights to produce an interpretive report. This report not only encapsulates the predictive analysis, but also enriches it with contextual intelligence, offering a multifaceted understanding of the target combination’s potential.

### Dataset collection and preprocessing

To train a machine learning model, we collected single-cell RNA-seq datasets of four different types of tumors to ensure the richness of the data (detailed in Table 1). We completed the preprocessing using Scanpy, which included: standardizing data formats from various sources, rigorous quality control to remove low-quality cells and contaminants, and normalization. We identified highly variable genes to understand cellular heterogeneity. Dimensionality reduction was performed through principal component analysis (PCA), followed by cell clustering and visualization to identify and illustrate cellular subpopulations. Finally, we analyzed gene expression patterns within these subgroups to elucidate their roles in the tumor microenvironment, informing the design of BsAbs drugs.

**Table 1:** Datasets summary

Tumor type	Cell number	Patient number
NSCLC	208506	58
Pancreatic cancer	308181	27
HNSCC	149609	18
Liver cancer	42762240	14

### Training dataset and pairwise label generation

In the construction of our machine learning model, the availability and richness of labeled data serve as critical determinants of the model’s performance and its capacity for generalization. However, the domain of target combination in BsAbs drug presents a unique challenge due to the relatively limited pool of BsAbs that have progressed to clinical stages, and an even smaller subset that has been approved. This scarcity of traditional pointwise supervised learning samples could potentially compromise the model’s training efficacy. To circumvent this limitation, we adopted an innovative approach by leveraging the clinical progression data of bispecific drugs. This strategy enabled us to enrich our training dataset through a pairwise comparison methodology. Specifically, we assigned higher model scores to drugs that have been approved over those undergoing clinical progress, and similarly, drugs in advanced clinical phases were scored above those in preliminary or preclinical stages. This pairwise scoring mechanism facilitated the utilization of available data more effectively, by transforming the inherently limited dataset into a richer, comparative training resource. We collected clinical progress data on 791 bispecific drugs targeting tumor indications (Supplementary Table 1). These bispecific drugs span across 5 different clinical phases, as detailed in Table 2.

**Table 2:** Summary of clinical progress

Number	Clinical progress
1	Approved, NDA/BLA
2	Phase 3, 2/3
3	Phase 1, 2
4	Preclinical, IND application
5	Discontinued/pending

### Feature engineering

To ensure the effectiveness and high accuracy of machine learning models in predicting target combinations of BsAbs, this study constructed features from multiple biological perspectives. The construction of all these biological features aims to comprehensively assess the potential anti-tumor effects and safety of BsAbs through different biological and data-driven methods. The construction of features relies on rich biological data, including gene expression data, pathway data, etc. These feature groups include:

#### Safety-based feature group

To assess the safety implications of using combined targets, we computed the harmonic mean of the differences in average gene expression between cancerous tissue and adjacent normal tissue. This calculation provides a nuanced safety score ${\text{score}}_{{\text{safe}}}$, with higher values indicating a preferable safety profile. The formula for this calculation is as follows, where *x* and *y* represent two targets:

$$d = {\text{expr}}_{{\text{tumor}}} - {\text{expr}}_{{\text{nomal}}}$$

$${\text{score}}_{{\text{safe}}} = \frac{d_x *d_y }{{d_x + d_y }}.$$

#### Target mechanism of action-based feature group

This feature group explores the synergistic potential of targets by analyzing their co-occurrence within shared biological pathways, including those cataloged in the Kyoto Encyclopedia of Genes and Genomes (KEGG) pathways and Gene Ontology (GO) annotations. By aggregating data on pathway affiliations from comprehensive databases, we calculate a feature score based on the frequency of gene pair co-occurrences within these pathways, providing insights into their collaborative mechanisms of action.

#### Gene embedding representation-based feature group

Drawing on the Gene2Vec (Du et al. 2019) methodology, akin to the Word2Vec model in natural language processing, gene embedding seeks to understand gene associations by representing each gene as a high-dimensional vector, revealing the complex regulatory relationships between genes. Through this approach, genes exhibiting similar expression patterns or functional characteristics are positioned proximately within the vector space. The Euclidean distance between gene vectors serves as a measure of similarity, offering a predictive gauge for the viability of combining specific targets based on their gene embedding representations:

$$d(x,y) = \sqrt {{\mathop \sum \limits_{i = 1}^n (x_i - y_i )^2 ,}}$$

where *x* and *y* are the vectors representing the two targets, and *n* is the dimensionality of the vectors.

#### Target activity-based feature group

This category of features was crafted to gauge the expression levels and the prevalence of positive gene expression within specific cell populations, thereby offering insights into the gene or target functions across various cell types within the tumor microenvironment. Key features within this group include:

*Double positive proportion of bispecific targets in cells*: This metric evaluates the incidence rate at which both targets exhibit positive expression within the same cell, serving as an indicator of the targets’ synergistic activity.

*Sum of the total positive proportions of each target in cells*: By calculating the aggregate of the proportions where each target is positively expressed in cells, this feature provides a measure of the overall target activity.

*Minimum of the total positive proportions of each target in cells*: This feature captures the lesser of the total positive proportions of the two targets, reflecting the combined activity level of the target pair.

To construct these features, we delved into single-cell transcriptome data, extracting target expression values specific to various cell populations. Our analysis particularly focused on cell populations pivotal to the tumor microenvironment, such as exhausted CD8^+^T cells, Tregs, macrophages, and epithelial cells. For each candidate gene, we calculated the average expression level across the most significantly expressing cell populations, identifying the two populations with the highest expression levels for further analysis. Subsequently, we determined the total positive proportion for each gene within these key cell populations, defining it as the fraction of cells exhibiting gene expression levels above a predefined threshold. Additionally, we assessed the double positive proportion for each gene pair, quantifying the proportion of cells where both genes surpassed the expression threshold. This threshold-based approach facilitated a rigorous quantification of gene activity, enabling a precise evaluation of potential bispecific target combinations.

The formula for calculating the total positive proportion ($P_{{\text{gene}}}$) for a gene is given by the ratio of cells within a specific cluster where the gene’s expression level (${\text{expr}}_{{\text{gene}},i}$) exceeds a predetermined threshold ($T$). Here, *N* represents the total number of cells within the cluster, and *I* is an indicator function that equals 1 when the gene’s expression in the *i*th cell is greater than *T*, and 0 otherwise:

$$P_{{\text{gene}}} = \frac{{\sum_{i = 1}^N I({\text{expr}}_{{\text{gene}},i} > T)}}{N}.$$

Moreover, the double positive proportion $(P_{{\text{gene}}1,{\text{gene}}2} )$ is calculated by identifying cells where both target genes are expressed above the threshold, defined by:

$$P_{{\text{gene}}1,{\text{gene}}2} = \frac{{\sum_{i = 1}^N I({\text{expr}}_{{\text{gene}}1,i} > T) \cdot I({\text{expr}}_{{\text{gene}}2,i} > T)}}{N}.$$

#### Target expression correlation-based feature group

We employ the Pearson correlation coefficient to quantify the linear relationship between the expression levels of two targets within single cells. This statistical metric elucidates the degree of correlation, where values near +1 or −1 indicate strong positive or negative correlations, respectively, and values close to 0 suggest a lack of linear association. Such insights are pivotal for understanding the cooperative or independent actions of targets, informing the strategic design of therapeutic interventions:

$$r = \frac{{\sum(x_i - \overline{x})(y_i - \overline{y})}}{{\sqrt {{\sum(x_i - \overline{x})^2 }} \sqrt {{(y_i - \overline{y})^2 }} }},$$

where $x_i$, $y_i$ are the expressions of two genes in different single cells, and $\overline{x},\overline{y}$ are the mean expressions of the two genes, respectively.

### Experimental settings

In this study, we embarked on a rigorous evaluation of various machine learning models to ascertain their efficacy in predicting target combinations. Utilizing a diverse array of engineered features, we contrasted the performance of seven distinct models: Logistic Regression (Yuan et al. 2022), Decision Tree, Random Forest (Fonseca et al. 2024), Gradient Boosting Classifier (GBDT), Deep Neural Networks (DNN) (Graves et al. 2020), XGBoost (Chen and Guestrin 2016) and XGBoost model with pairwise learning. The configuration of these models was meticulously optimized, with parameters set to ensure both accuracy and efficiency. The Decision Tree Classifier was adjusted with a minimum leaf node count of 5 and a maximum depth of 10, while the Logistic Regression model was fine-tuned with an L2 regularization coefficient of 0.1 and set to iterate up to 1000 times. The Random Forest Classifier was deployed with 100 trees, and the Gradient Boosting Classifier was calibrated with a 0.1 learning rate alongside 100 trees. The DNN was designed with five fully connected layers (256, 128, 64, 32, and 1), using LeakyReLU activation and Dropout for overfitting mitigation, and optimized with Nadam. The XGBoost models, both in their pointwise and pairwise variations, were set with a learning rate of 0.1, a 100-tree ensemble, and a maximum depth of 10.

The pairwise adaptation of the XGBoost model employs a loss function that evaluates the predictive accuracy based on the relative rankings of target pairs, thereby enhancing the model’s capacity to optimize the order among target pairs. This method stands in contrast to the traditional pointwise technique. Traditional pointwise technique assesses each target pair’s predictive value in isolation, without consideration of their interrelations. By prioritizing the relative ranking of target pairs, our study effectively expands the usable training samples.

To ensure the robustness and generalizability of our models, we implemented a fivefold cross-validation strategy, thereby bolstering the reliability of our results across varied dataset partitions. AUC served as the principal metric for evaluating model performance, providing a comprehensive measure of model accuracy.

### Integration of GPT with retrieval argument generation

This study combines the output results of machine learning models with the LLM model through a retrieval argument approach, fully leveraging the rich pre-trained knowledge within the LLM model and the accuracy of the machine learning model to produce bispecific drug target design analysis results with stronger interpretability. GPT-4 was chosen as the optimal LLM model for this task. To enable the GPT-4 model to better understand the basis of the machine learning model’s predictions, we discretized the most important features used during the machine learning prediction process into up to five equal-frequency bins. Specifically, the discretization process involves dividing the range of each feature into several intervals with an equal number of observations and converting the features into different natural languages based on the size of the feature values.

When querying whether two targets can be combined, we first retrieve the natural language results of the discretized features of these two targets and include the final result of the machine learning prediction in the prompt provided to the GPT-4 model. This retrieval-enhanced approach allows the GPT-4 model to supplement more arguments for the result discussion based on the machine learning model’s prediction results. At the same time, we also provided textual explanations for the meanings of these features. In addition to utilizing the rich pretrain knowledge of the GPT-4 model, we also stimulated its ability to invoke search engines to obtain the latest target research results by tuning the prompt. The complete prompt is displayed in Supplementary Data 1. Ultimately, GPT-4 outputs the final report by integrating the important features of machine learning, the prediction results of the machine learning model, the LLM’s own pre-trained knowledge, and the latest research progress on the target retrieved from search engines. Compared to the probabilistic results of the machine learning model alone, this approach of combining probabilities, important features, and rich arguments can better assist drug designers in making informed decisions. The entire framework, which includes feature calculation, model prediction, important feature identification, and prompt engineering, is referred to as BSPAI, and the results section showcases the output results of BSPAI after a combined retrieval of CD274 and CTLA4. Supplementary Data 2 shows the machine learning results for CD274 and CTLA4 target pair.

## Results

### Feature ablation study

In our exploration to decipher the contribution of individual feature groups to the prediction accuracy of target combinations for BsAbs, we embarked on a series of feature ablation studies. These experiments were methodically designed to sequentially incorporate each feature group into the model, followed by its retraining and evaluation to monitor the resultant variation in performance metrics. The outcomes of these experiments, depicted in Table 3, illuminated the critical influence of diverse biological features on augmenting the model’s predictive efficacy.

**Table 3:** Results of feature ablation study

Features	AUC (%)	Improvement (%)
Double positive proportion	54.76	–
+ safety-related features	67.06	12.3
+ mechanism analysis features	71.74	4.68
+ gene embedding features	73.72	1.98
+ target expression of single cell	86.19	12.47
+ grouped cell type expression features	89.29	3.1

Initially, employing the double positive proportion feature alone yielded an AUC of 54.76%, underscoring the predictive value of the double positive proportion. The results also reveal the limitations in predicting cases where the two targets are not expressed on the same cell. The subsequent inclusion of safety-related features marked a significant leap, boosting the AUC to 67.06%, with a substantial improvement of 12.3%. This enhancement accentuates the criticality of safety considerations within the drug discovery process and the prediction of treatment outcomes. Further refinement was achieved by adding mechanism analysis features, leading to a 4.68% increase in AUC, reaching 71.74%. This increment highlights the importance of shared biological pathways among targets. The incorporation of gene embedding features resulted in a modest yet meaningful increase in AUC by 1.98%, achieving 73.72%, indicating the utility of capturing complex gene relationships through embedding techniques. The notable surge in predictive accuracy came with the target expression of single cell, which propelled the AUC to 86.19%, an impressive rise of 12.47%. This leap signifies the fundamental importance of target expression levels of single cell in determining the potential success of bispecific drug combinations. This reflects the advantages of single-cell transcriptomics sequencing technology, providing unprecedented levels of detail for understanding cell behavior, intercellular interactions, and complex biological processes. Especially in the study of the tumor microenvironment, tumor tissue is not composed of a single cell type, but is a complex ecosystem containing multiple cell types (such as tumor cells, immune cells, endothelial cells, etc.). Single-cell transcriptomics can reveal the gene expression characteristics of these different cell types, as well as their unique roles in tumor development and treatment response. Finally, integrating grouped cell type expression of single cell features further refined the model’s accuracy, culminating in an AUC of 89.29%, an increase of 3.1%. This final improvement emphasizes the necessity of analyzing target expressions within specific single cell types, particularly for targets such as PD-1 and CTLA4, which exhibit pronounced expression predominantly within T cells. This part of the research highlights the importance of grouped cell type expression features of single cell, meaning that the analysis of gene target expression needs to be conducted within specific cell populations, rather than a generalized analysis across all cell types.

The incremental feature integration strategy employed in this study not only methodically enhanced the model’s predictive accuracy, but also highlighted the multifaceted nature of factors influencing the success of bispecific target drugs, from safety and biological mechanisms to complex gene interactions and expression dynamics.

**Fig. 2:**
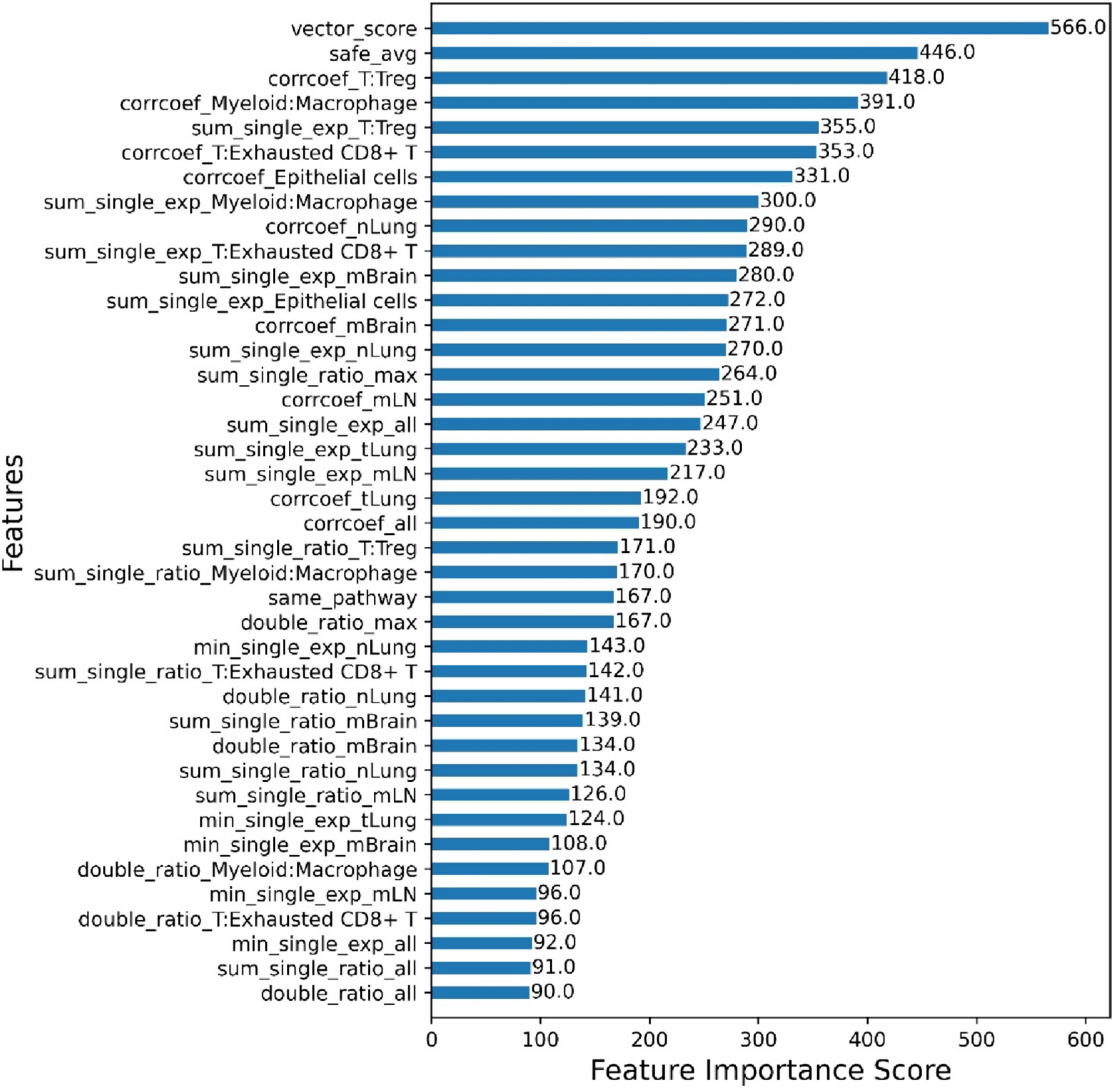
Feature importance. The vertical axis represents the names of various features, ranked according to their importance in the predictive model, with importance decreasing from top to bottom. The horizontal axis displays the “feature frequency” scores of each feature within the XGBoost model, reflecting the frequency of features acting as split nodes in the decision tree ensemble, thereby indicating their relative importance in the model’s decision-making process. The bar chart visually presents the top 40 features and their importance scores

In our quest to unravel the hierarchical significance of features within the predictive modeling process, we embarked on the feature importance analysis. Leveraging the XGBoost model, we utilized the “feature frequency” method, which quantifies the importance of a feature based on its recurrence as a split node throughout the ensemble of decision trees. This metric provides a clear indication of a feature’s relevance, with those frequently serving as pivotal points for decision splits across multiple trees deemed most critical. Supplementary Table 2 shows the meaning of each feature. Figure 2 reveals that target similarity, as deduced through gene embedding techniques, emerged as the paramount feature, underscoring its vital role in model predictions. Gene embedding draws inspiration from advances in natural language processing, utilizing the concept of gene co-expression to simulate gene interactions, mirroring the way words associate in context within natural language processing. It allows embedding models to capture the “context” of genes, akin to word co-occurrence, thereby understanding gene expression patterns and their interactions in high-dimensional spaces. By transforming genes into numerical vectors, gene embedding encapsulates information from gene co-expression networks, offering robust support for uncovering gene functions, disease associations, and potential therapeutic targets. This was closely followed by the safety_score, which encapsulates the potential risk factors associated with the bispecific targets. Excellent BsAb targets require tumor expression specificity to reduce the risk of adverse event (AE) due to on-target off-tumor effects. The safety feature is also consistent with the laws of clinical practice, where the primary endpoints in a Phase I clinical trial are usually safety and initial efficacy. It will be further advanced to Phase II/III efficacy validation studies, subject to ensuring safety.​

The Pearson correlation coefficient between the two target genes, indicative of the concordance of their expression levels, ranked third in importance. The co-expression feature suggests that the biological nature of the synergistic effect of BsAb is derived from the co-expression of target pairs and the overlap or co-amplification of downstream signaling pathways. For example, AK104, which has been successfully approved, primarily targets PD-1 and CTLA4 co-expression on exhausted CD8^+^T, and doubly blocks PD-L1/2 and CD80/86 to inhibit downstream signaling pathways, thereby superimposing stimulated T secretion of IFNγ to kill tumors. In addition, CTLA4 pathway can also significantly enhance the secretion of IL-2 by CD4^+^T cells and promote the proliferation, survival and tumor killer activity of T, NK and other immune cells in the tumor microenvironment. Additionally, the single positive fraction of targets within T cells was identified as a key determinant, highlighting the significance of target activity within T cells. The single-expression feature reminds developers of the high heterogeneity of target expression in the tumor microenvironment. Again, taking AK104 as an example, in addition to targeting co-expressed CD8^+^ T cells, one arm of AK104 can also target single expressed PD-1^+^CD8^+^T or single-expressed CTLA4^+^ CD4^+^T cells, which are abundant in the tumor microenvironment and exert optimal anti-tumor activity by covering more effector T cells.

### Performance and model comparison

**Fig. 3:**
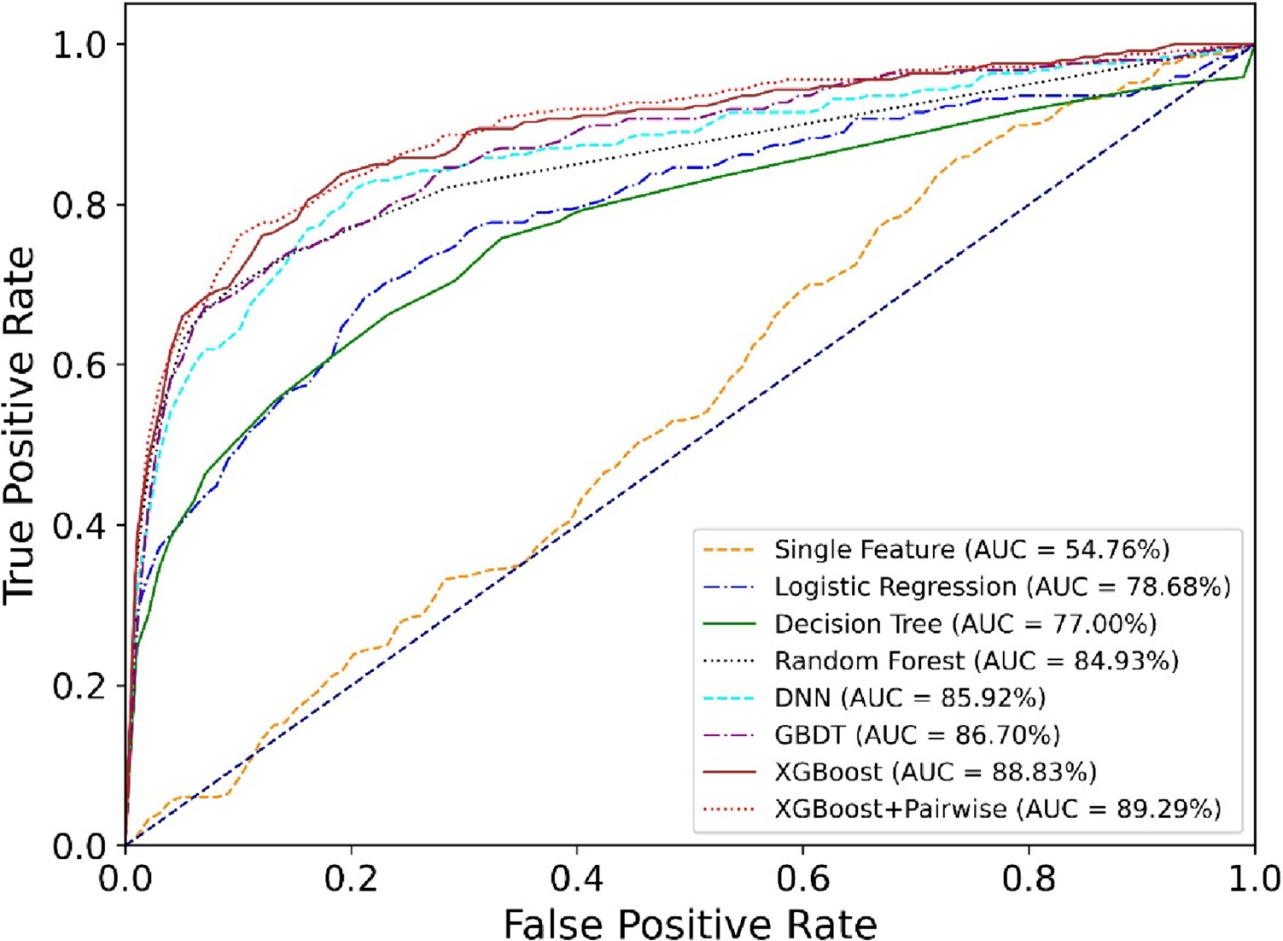
ROC curves of various models (taking NSCLC as an example).  The receiver operating characteristic (ROC) curve is a graphical plot that illustrates the performance of a binary classification model by displaying the trade-off between the true positive rate (sensitivity) and the false positive rate (1 − specificity) across a range of thresholds. The AUC metric quantifies the overall ability of the model to discriminate between the two classes, with higher values indicating better performance

In this study, we evaluated the efficacy of seven distinct machine learning models to predict the viability of target combinations for BsAbs (Fig. 3). Our investigation commenced with an examination of model performance using a single feature for assessment. Drawing from extensive drug design expertise, we selected “double positive proportion” as our feature of interest, which yielded an AUC of 54.76%. This result suggests a base performance level, underscoring the feature’s limited applicability primarily to scenarios where both targets are simultaneously highly expressed within the same cell type. This limitation became apparent in need of bridging effect cell to target cells (like CD3 and CLDN18.2), necessitating a broader evaluative framework incorporating multiple features for a holistic assessment. Besides, using only the double positive proportion as feature, performance can be unstable across different types of cancer data (Table 4).

**Table 4:** Performance comparison of five-fold cross-validation in different types of cancer

Model name	AUC (NSCLC) (%)	AUC (pancreatic cancer) (%)	AUC (HNSCC) (%)	AUC (liver cancer) (%)
Single feature	54.76	53.35	49.38	46.60
Logistic regression	78.68	76.81	76.21	76.43
Decision tree	77.00	77.18	76.67	73.38
Random forest	84.93	84.46	83.29	84.22
DNN	86.08	80.78	82.08	79.69
GBDT	86.70	85.36	85.37	86.19
XGBoost	88.83	88.04	88.55	87.61
XGBoost + pairwise	89.29	88.04	88.93	87.79

*NSCLC* non-small cell lung cancer, *HNSCC* head and neck squamous cell carcinoma

After optimizing machine learning models such as logistic regression and decision trees, we observed a significant improvement in predictive accuracy compared to the single-feature method. Specifically, Logistic Regression demonstrated a significant uplift, achieving an AUC of 78.68%. This enhancement highlights the inherent advantage of machine learning models over traditional statistical methods reliant on a single feature—machine learning models integrate multifaceted insights to derive a comprehensive conclusion. The application of ensemble learning methods, which amalgamate insights from various sub-models including Random Forest, GBDT, and XGBoost, marked a further leap in performance. The DNN model yielded an AUC of 86.08%, placing it between Decision Trees and GBDT models. This outcome underscores the challenges in training deep neural networks with limited positive samples and highlights the effectiveness of ensemble methods in this context. Notably, the XGBoost model, employing a pointwise approach with a cross-entropy loss function, attained an impressive AUC of 88.83%. This progression underscores the efficacy of ensemble methods in enhancing predictive accuracy beyond that of single model approaches. The pinnacle of our model comparison was reached with the integration of the XGBoost model utilizing a pairwise learning approach, culminating in an optimal AUC of 89.29%. This outcome validates our hypothesis that considering the ordinal relationships between different clinical stages—particularly in contexts characterized by a scarcity of bispecific drug clinical samples—significantly enhances model performance. The pairwise approach, by prioritizing the relative ranking among target pairs, emerges as a potent methodology in scenarios marked by limited positive samples, offering a promising avenue for application in similar research contexts. Table 4 demonstrates that our model exhibits high predictive accuracy across a variety of cancer types, not limited to NSCLC. This suggests the potential for incorporating datasets from additional cancer types in the future, enabling a comprehensive pan-cancer analysis.

This comparative analysis not only delineates the superior performance of machine learning techniques over traditional single-feature statistical methods, but also exemplifies the potential of considering ordinal relationships in enhancing predictive accuracy for target combinations in bispecific antibody.

### Discretization for enhanced GPT integration

To enable the GPT model to make more matched argumentative explanations for the output results of the machine learning model, we converted the features used by the XGBoost model during the prediction process into nature language and passed them to the GPT model. Due to the context text length limitation of the GPT model, we selected the six most important and representative features based on the aforementioned feature importance, along with other various types of information, to be communicated to the GPT model.

We adopted a discretization strategy for continuous numerical features, segmenting them into intervals to render them more comprehensible from a natural language processing standpoint. The outcomes of this discretization, translating numerical data into natural language segments, are presented in Table 5. This approach not only accentuates the contributions of individual features to the model’s predictive success, but also exemplifies the integration of machine learning insights with natural language processing capabilities. Through this methodology, we aim to provide a nuanced understanding of the model’s decision-making process, thereby facilitating the development of more interpretable and actionable insights in the realm of bispecific drug design.

**Table 5:** Results of feature discretization into natural language

Gene embedding similarity	Safe score	Pearson correlation coefficient	Single_ratio_max	Double_ratio_max
Very similar (0–3.67)	Unsafe (−∞ to −0.0071)	Strongly negative correlation (−∞ to −0.020)	Low (0–0.11)	Low (0–0.00019)
Somewhat similar (3.67–4.02)	Moderately low safety (−0.0071 to −0.000073)	Weakly negative correlation (−0.020 to −0.004)	Moderately low (0.11–0.24)	Moderately low (0.00019–0.0015)
Similar (4.02–4.34)	Safe (−0.000073 to 0.0012)	Uncorrelated (−0.004 to 0.004)	Moderate (0.24–0.45)	Moderate (0.0015–0.0071)
Dissimilar (4.34–4.77)	Moderately high safety (0.0012–0.013)	Weakly positive correlation (0.004–0.020)	Moderately high (0.45–0.67)	Moderately high (0.0071–0.35)
Very dissimilar (4.77 to +∞)	High safety (0.013 to +∞)	Strongly positive correlation (0.020 to +∞)	High (0.67–1)	High (0.35–1)

### Target prediction results

Table 6 shows the list of the top 3 target pairs in each type of BsAbs, ranked from high to low according to the predicted probability of bispecific target pairs being marketed. Supplementary Table 3 displays the top 100 target pairs ranked from high to low according to the predicted probability of bispecific target pairs being marketed. Since CD3 (CD3E) targets T cells and is a universal type of T cell activator, only the top two pairs for all CD3 combinations are shown.

**Table 6:** Top3 target pairs of each type BsAbs

Type	Gene1	Gene2	Predict score	Clinical stage
Bridge two cells	CD40	EGFR	0.89	0
	CD3	ERBB3	0.84	0
	CD274	CTLA4	0.79	4
TAA+TAA	CD47	ERBB2	0.69	4
	EGFR	MUC1	0.61	2
	EGFR	ERBB2	0.57	0
Tumor immunotherapy targets	CTLA4	ICOS	0.67	0
	PDCD1	TNFRSF9	0.66	4
	LAG3	PDCD1	0.58	4

CD274+CTLA4 represents an important target pair currently under clinical investigation in Table 6. In various cancers, including NSCLC and melanoma, immune checkpoint receptors such as PD-1 and CTLA4 are highly co-expressed on immune cells like exhausted CD8^+^ T cells. The upregulation of CTLA4 expression is a key mechanism of PD-1 resistance. Ligands CD80 and CD274 are co-expressed on antigen presentation cells. Combination therapy with PD-1 and CTLA4 monoclonal antibodies (e.g., nivolumab combined with ipilimumab) has been approved, though it has been observed to synergistically increase side effects such as colitis. BsAbs maintain efficacy while potentially improving safety through mechanisms such as reduced CTLA4 activity and specifically targeting the tumor microenvironment. For instance, AK104 (PD-1+CTLA4) has been approved for platinum-refractory or metastatic cervical cancer, and KN046 (CD274+CTLA4) has entered Phase III clinical trials for pancreatic ductal adenocarcinoma (PDAC) and NSCLC. The BsAbs targeting CD274+CTLA4 operates with one arm targeting CD274 and the other targeting CTLA4. Its mechanism of action includes: (1) releasing immune suppression on effectors like CD8^+^T cells expressing both (approximately 28–43%) and single (2–10%) PD-1/CTLA4, preventing resistance; (2) significantly aggregating CTLA4 receptors through CD274 side cross-linking to synergistically relief exhausted CD8^+^T suppression; (3) both arms can bridge effector cells, pulling CD8^+^T cells closer to the tumor, or bridge CD4^+^T and DC cells to enhance immune synapse formation and antigen presentation; (4) utilizing an IgG1 mutant subtype with reduced antibody-dependent cell-mediated cytotoxicity (ADCC), antibody-dependent cell-mediated phagocytosis (ADCP) and complement dependent cytotoxicity (CDC) functions, minimizing the risk of NK or tumor-associated macrophages (TAM)-mediated T cell clearance. Therefore, the predicted target pair of CD274+CTLA4 has high druggability, which proves the reliability of our model.

CD40+EGFR or CD3+ERBB3 target pairs belong to the category of bridging effector cell and target cells, and no BsAbs drugs are currently in clinical trials in Table 6. ERBB2, ERBB3 and EGFR, all members of the human epidermal growth factor receptor family, function as transmembrane proteins that, upon binding with growth factors, undergo dimerization to mediate signal transduction and regulate cell growth, division and repair. These processes are intimately linked to the development of numerous cancers. A variety of anticancer drugs targeting EGFR and ERBB2 have been approved, patritumab–deruxtecan (antibody–drug conjugate targeting ERBB3) also entered phase III clinical trials currently. The results demonstrated promising clinical activity of patritumab–deruxtecan across various subgroups of breast cancer patients in phase I/II clinical trial, with objective response rate (ORR) reach to 36.2%, and median overall survival (OS) of 13.7 months. Therefore, ERBB2/3 and EGFR are ideal tumor-associated antigens, which are suitable for binding with T and B cell activators such as CD3 and CD40 agonists to construct bispecifics, and kill tumor cells by bridging immune cells to tumor. Currently, there are no CD3+ERBB3 or CD40+EGFR BsAbs drugs in clinical stages or disclosed to be in preclinical research. Some studies have revealed that upregulation of ERBB3 is a key resistance mechanism to EGFR and ERBB2 targeted therapies (Kruser and Wheeler 2010; Leto et al. 2015; Zhang et al. 2020), highlighting the importance of developing novel therapeutic strategies targeting ERBB3.

ERBB2+EGFR constitutes a TAA+TAA type target pair, for which, currently, no BsAbs drugs have entered clinical trials (Table 6). Currently, several anticancer mAb drugs targeting EGFR or ERBB2 have been approved. For instance, monoclonal antibodies targeting ERBB2, such as trastuzumab and pertuzumab, are approved to treat ERBB2-positive breast cancer. Similarly, cetuximab, which targets EGFR, has been authorized for treating metastatic colorectal cancer and head and neck cancer, among others. Additionally, various small molecule inhibitors targeting EGFR and/or ERBB2 have been approved for treatment of cancers such as NSCLC. In cancers such as NSCLC, EGFR and ERBB2 are highly co-expressed and approximately 10–15% of patients developing resistance to EGFR-targeted therapies exhibit ERBB2 mutations. Anticipating the development of such BsAbs, targeting both EGFR and ERBB2 signaling pathways could not only synergistically inhibit tumor growth and enhance anticancer efficacy, but also effectively prevent resistance caused by mutations in either target.

### Complete analysis report

After inputting the two targets CD274 and CTLA4 into our model BSPAI, the output of a complete analysis report is as follows:

The analysis of CD274 (PD-L1) and CTLA4 as dual targets for the design of a bispecific antibody drug takes into account their individual characteristics, clinical progress in monoclonal antibody development, and potential for combined treatment efficacy based on machine learning model features and external literature.

#### CD274 (PD-L1)

*Clinical progress*: PD-L1, targeted by antibodies like durvalumab and socazolimab, has shown varied clinical efficacy across different cancer types. For instance, in metastatic breast cancer, durvalumab did not significantly improve overall survival (OS) in the overall population but showed promise in triple-negative breast cancer (TNBC) patients, especially those with CD274 gain/amplification. Another study on socazolimab for recurrent or metastatic cervical cancer demonstrated its safety and efficacy, with a median OS of 14.72 months and an objective response rate (ORR) of 15.4%. These results underscore the potential of PD-L1 inhibitors in certain cancers, suggesting a role for CD274 as a target in bispecific antibody development.

#### CTLA4

*Clinical advances*: CTLA4 inhibitors, such as ipilimumab, have established efficacy in melanoma, with a reported 5-year survival rate of 18.2%. PD-1 inhibitors, targeting PD-L1, have been found to be more effective and less toxic than CTLA4 inhibitors in melanoma, indicating the importance of the PD-1/PD-L1 pathway in immunotherapy.

#### Machine learning model analysis

- Gene2vec score: medium. This suggests a moderate level of similarity and potential functional or pathway overlap between CD274 and CTLA4.
- Dual target expression double-positive percentage score: above average. This indicates a significant portion of cells expressing both targets, which is promising for a bispecific antibody’s effectiveness.
- Target safety score: low. This presents a challenge, suggesting potential safety concerns due to the expression of these targets in non-cancerous tissues.
- Target correlation score: below average. This indicates a less than ideal correlation in their expression, which might affect the dual targeting strategy’s efficiency.
- Pathway enrichment analysis score: same. This suggests that CD274 and CTLA4 function within the same pathway, supporting the rationale for their combined targeting.
- Machine learning model prediction score: 0.93. Ranked 4 out of 24,753 candidates, highlighting a high potential for clinical efficacy in combining CD274 and CTLA4 targeting.

#### Conclusion

The combination of CD274 and CTLA4 as targets for bispecific antibody drugs is supported by individual successes in monoclonal antibody therapies and the promising machine learning model prediction score. The medium gene2vec score, above-average dual target expression, and shared pathway involvement underscore their potential synergy. However, the low target safety score and below-average target correlation score present challenges that need to be addressed.

Given the efficacy of PD-L1 and CTLA4 inhibitors in various cancers, especially in melanoma and certain subsets of breast and cervical cancers, a bispecific antibody targeting both CD274 and CTLA4 has a strong rationale. The key will be to navigate safety concerns and ensure that the bispecific antibody can effectively engage both targets to improve therapeutic outcomes. Further research and clinical trials will be crucial to explore the full potential and address the challenges of this innovative therapeutic approach.

## Discussion

This study introduces a pioneering approach that seamlessly integrates machine learning and GPT techniques to advance the design of BsAbs drugs, a crucial area in drug design that necessitates the identification of effective target combinations. Our methodology commenced with constructing pairs of targets from a curated list of hundreds of common membrane proteins. This study demonstrates the following advantages in addressing similar problems:

1. Depth optimization of feature engineering: We have deeply optimized feature engineering, including multidimensional features based on target activity, safety, type, pathway mechanisms, and gene embedding representations, which are more complex and comprehensive than the simpler feature sets.
2. Pairwise advantages: In the context of selecting target combinations for BsAbs, the number of bispecific therapeutics that have progressed to clinical trials remains relatively small, and even fewer have reached the market. Consequently, traditional pointwise supervised learning approaches may encounter challenges due to a scarcity of samples. To address this, we leveraged the clinical development stages of BsAbs by assigning progressively decreasing model scores to drugs based on their approval status and clinical phase progression, from approved to Phase III, II, I, and preclinical stages. This strategy allows for training with a comparatively abundant dataset by utilizing a pairwise comparison approach, thereby enhancing the model’s learning potential in the context of limited sample availability.
3. Enhancement of interpretability with GPT: This is a significant departure from most previous machine learning methods, which often lack sufficient interpretability, whereas understanding the reasons behind model predictions is crucial in drug design.

However, this study also has some limitations. This study is limited to the BsAbs, with analyses of tri- or tetra-specific antibodies involving three or four targets, respectively, not yet explored. Future endeavors could extend and apply similar biological feature-based approaches to these more complex multi-specific antibody. In future work, our research will continue to expand and optimize the combined approach of machine learning and GPT technology to further improve the efficiency and accuracy of bispecific antibody drug design. Specific future directions include:

1. Expansion and diversification of the dataset: We plan to expand and diversify the current target dataset used, including a wider variety of membrane-expressed targets, drug clinical data, single cell transcription data of more tumors, and proteomics data. This will not only improve the generalization ability of the model, but also help explore more potential bispecific target combinations.
2. Further optimization of algorithms and feature engineering: XGBoost performed excellently in this study, but the DNN model did not meet expectations. Compared to tree models such as decision trees and XGBoost, DNNs are more sensitive to datasets with a smaller number of positive samples, leading to suboptimal training outcomes. Despite the provision of rich biological features, DNNs did not show a clear advantage in extracting non-linear relationships from the data, which might be due to tree models being more effective in processing these types of features and less prone to overfitting. Training DNNs requires substantial data and computation, along with meticulous network design and parameter tuning. Even though mainstream optimization techniques and overfitting prevention strategies were employed, DNNs still struggled to surpass the XGBoost model under conditions of limited data. In the future, as more and more BsAbs are approved, there will be more opportunities to experiment with deep learning models, especially in integrating antibody prediction models with LLMs through an end-to-end training approach. At the same time, we aim to conduct a more in-depth analysis and optimization of feature representation to more accurately reflect the characteristics and interactions of targets.
3. In-depth study of model interpretability: Although GPT techniques have been used to explain the effectiveness of bispecific target combinations, we plan to further deepen this area in the future. By collecting data such as multi-antibody project reports, the LLM model is fine-tuned to further enhance its interpretability in the field of bioinformatics.
4. Experimental validation and preclinical studies: Laboratory validation of high-scoring bispecific target combinations predicted by the model, as well as conducting preclinical studies to further confirm the efficacy and safety of these combinations. Collaborate with experts in the fields of molecular biology and clinical research to leverage their expertise and techniques to improve the overall quality and success rate of drug design.

Gene embedding methods can capture more complex inter-gene relationships, and such features rank first in feature importance rankings, suggesting that drug development researchers should also focus on the gene interaction networks between two targets during drug design. We also found that data on the positive proportion of different cell types obtained from single-cell data can significantly improve performance, indicating that single-cell data, by grouping cell types, can provide a finer understanding of the expression and mechanisms of targets in various cell types within the tumor microenvironment more finely than bulk-RNA seq. Safety features are another important consideration. If the safety score is low, careful consideration must be given to the drug design, even if expression and other indicators suggest high efficacy. For instance, safety issues can be collectively addressed by reducing the affinity between the antibody and antigen or by reducing the dosage during clinical design. Through our efforts, we hope to provide a stronger, more accurate and more interpretable tool for the design and development of BsAbs, thereby helping to accelerate the research and development process of new drugs and bring more treatment options to patients.

## Conclusion

By integrating the XGBoost model with pairwise learning and GPT technology, we have improved the prediction accuracy of target combinations in BsAbs to 89.28%. This provides researchers with highly reliable guidance. Additionally, combining it with GPT enhances interpretability, facilitating bispecific drug development researchers to understand and make accurate target combination selections more effectively.

The abbreviations involved in this article are listed in Supplementary Table 4.

## Supplementary Information

Below is the link to the electronic supplementary material.

Supplementary file1 (DOCX 16 KB)

Supplementary file2 (DOCX 12 KB)

Supplementary file3 (XLSX 64 KB)

Supplementary file4 (XLSX 97 KB)

Supplementary file5 (XLSX 90 KB)

Supplementary file6 (XLSX 11 KB)

## Data Availability

The dataset is available on GSE131907 (Kim et al. 2020), GSE205013 (Werba et al. 2023), GSE164690 (Kurten et al. 2021), GSE156625 (Sharma et al. 2020).
