# Supplementary material for: BiSpec Pairwise AI: guiding the selection of bispecific antibody target combinations with pairwise learning and GPT augmentation
Source: J Cancer Res Clin Oncol. 2024 May 7;150(5):237. doi: 10.1007/s00432-024-05740-3 (PMC11076393; doi:10.1007/s00432-024-05740-3)
Supplement: Supplementary file 1 — Supplementary file1 (DOCX 16 KB) [file 432_2024_5740_MOESM1_ESM.docx]

Your task is to analyze whether two targets are suitable fwor the design of a bispecific antibody drug and more likely to achieve clinical efficacy. Conclude and provide the basis for your analysis.

Dual targets names: [{gene1}, {gene2}].

First, let's show some examples of the output:

Example: [ERBB2, EGFR]

ERBB2 and EGFR both belong to the human epidermal growth factor receptor family. As transmembrane proteins, they dimerize upon ligand binding, mediating cell growth, division, and repair, and are closely related to the development of various cancers. Currently, multiple antitumor drugs targeting EGFR or ERBB2 have been approved for marketing. For example, drugs targeting ERBB2, such as trastuzumab and pertuzumab, are used for the treatment of ERBB2-positive breast cancer, while drugs targeting EGFR, such as cetuximab, have been approved for the treatment of metastatic colorectal cancer and head and neck cancer, among others. Additionally, various small molecule inhibitors targeting EGFR and/or ERBB2 have been approved for use in tumor types such as NSCLC. EGFR and ERBB2 are highly co-expressed in tumor types such as NSCLC, and among patients who develop resistance to EGFR treatment, approximately 10% to 15% have ERBB2 mutations. However, there are currently no bispecific antibodies targeting both EGFR/ERBB2. It is anticipated that targeting both EGFR and ERBB2 signaling pathways simultaneously could not only synergistically inhibit tumor growth and improve anticancer efficacy but also effectively prevent resistance caused by mutations in one of the targets.

The analysis of ERBB2 and EGFR as dual targets for the design of a bispecific antibody drug involves several machine learning features, each providing a unique perspective on the potential success of these targets in clinical applications:

- Gene2vec Score: Reflects the similarity between genes based on gene expression data. For ERBB2 and EGFR, a "Low" score indicates that, according to the gene2vec model, these genes are not closely related in the genetic vector space. This might suggest divergent functions or pathways, potentially complicating their simultaneous targeting.

- Dual Target Expression Double-Positive Percentage Score: A "High" score here suggests a significant proportion of cells express both ERBB2 and EGFR. This is crucial for a bispecific antibody's efficacy, as it indicates a good target population within diseased tissues.

- Target Safety Score: A "High" safety score indicates that the difference in expression levels between cancerous and adjacent non-cancerous tissues is significant, suggesting targeting these genes could minimize effects on healthy cells, enhancing safety.

- Target Correlation Score: A "High" score suggests a strong positive correlation in the expression of ERBB2 and EGFR, indicating that when one is expressed, the other likely is too. This supports the concept of dual targeting with a bispecific antibody.

- Pathway Enrichment Analysis Score: A score indicating "Same" suggests both targets function within the same pathway. This could mean that targeting both could have a synergistic effect, potentially leading to a more effective therapeutic response.

- Machine Learning Model Prediction Score: Although not explicitly provided, this score, calculated by an XGBoost pairwise rank model, would integrate the above features to predict the likelihood of clinical success. Higher scores would indicate a greater potential for effective therapeutic outcomes.

Integrating these insights with the machine learning analysis, which includes gene2vec score, dual target expression double-positive percentage score, target safety score, target correlation score, pathway enrichment analysis score, and the final model prediction score, a comprehensive picture emerges. Despite a low gene2vec score indicating dissimilarity, the high scores in dual target expression, target safety, target correlation, and pathway enrichment alongside a model prediction score of 0.85 suggest a strong potential for clinical efficacy in combining ERBB2 and EGFR targets for bispecific antibody therapy.

Conclusion: These findings, combined with the clinical success of individual monoclonal antibodies targeting these proteins and the advanced rank in machine learning predictions, suggest that a bispecific antibody targeting both ERBB2 and EGFR could offer a promising therapeutic strategy. However, the challenges in developing such a therapy include overcoming resistance mechanisms, managing potential toxicities due to the broad expression of EGFR in normal tissues, and ensuring the bispecific antibody can effectively engage both targets to achieve a synergistic therapeutic effect. Continued research and clinical trials will be essential to fully understand the potential and limitations of this innovative therapeutic approach.

------------

End of examples.

Let's begin

You need to follow these steps in your thought process:

Step one. Introduction:

First, use a search engine to collect information related to these two targets, [{gene1}, {gene2}], summarize their respective characteristics, the clinical progress of their corresponding monoclonal antibodies, and the latest clinical data on ORR and OS. Also, check if there is any content related to the design of bispecific antibodies.

Step two. Reference machine learning features and their model analysis results,

Here are the features used by the machine learning model, with specific meanings for each feature:

- Gene2vec score: This value is generated by a pre-trained gene2vec model. Gene2Vec is a method for analyzing gene expression data, inspired by the Word2Vec model in natural language processing. Similar to how Word2Vec learns semantic relationships between words, Gene2Vec learns similarities and associations between genes. It represents each gene as a high-dimensional vector and learns these vectors through training data so that similar genes are close to each other in the vector space. The scores are divided into five levels: [very similar; somewhat similar; similar; dissimilar; very dissimilar].

- Dual target expression double-positive percentage score: This provides the proportion of cells expressing both targets in the dual target pair, divided into five levels: [low; lower-middle; middle; upper-middle; high].

Target safety score: This represents the harmonic mean of the difference in expression levels of the two targets in cancerous and adjacent non-cancerous tissues, calculated separately for tLung (early cancer tissue in NSCLC patients) and nLung (adjacent non-cancerous tissue in NSCLC patients), to assess the safety of the targets; a higher value indicates better safety. The scores are divided into five levels: [unsafe; lower-middle safety; safe; upper-middle safety; high safety].

- Target correlation score: Calculates the Pearson correlation coefficient of the expression of the two targets in single cells, divided into five levels: [strongly negatively correlated; weakly negatively correlated; uncorrelated; weakly positively correlated; strongly positively correlated].

- Pathway enrichment analysis score: This indicates whether the two targets function in the same pathway, divided into three levels: [no; possibly; yes].

- Machine learning model prediction score (important basis): An xgboost pairwise rank model was trained using currently marketed or clinically tested dual-target drugs to predict the success rate of marketing for the combined use of two targets. The score ranges from 0 to 1, with higher values indicating a higher likelihood of achieving clinical efficacy.

Step three. Integrate evidence, refine thinking:

Combine the information collected in step 1 with the following, including the specific values of the machine learning model features mentioned in step 2 and the results of the machine learning model predictions:

{ml_result}

When a particular aspect's score is low, use a search engine to collect relevant literature for verification.

Step four. Conclusion:

Based on the above analysis and collected evidence, summarize whether this pair of targets has potential for application in the design of bispecific antibody drugs and the challenges they may face.

Now, please directly output a paragraph, integrating the analysis results, external data, and thoughts to conclude whether [{gene1}, {gene2}] have potential for combined treatment with bispecific antibody drugs. Please incorporate external literature as much as possible, along with the above machine learning analysis results, to produce the final outcome.
