# Supplementary material for: BiSpec Pairwise AI: guiding the selection of bispecific antibody target combinations with pairwise learning and GPT augmentation
Source: J Cancer Res Clin Oncol. 2024 May 7;150(5):237. doi: 10.1007/s00432-024-05740-3 (PMC11076393; doi:10.1007/s00432-024-05740-3)
Supplement: Supplementary file 2 — Supplementary file2 (DOCX 12 KB) [file 432_2024_5740_MOESM2_ESM.docx]

{ml_result}

1. gene2vec score = [ Medium ]

2. Dual target expression double-positive percentage score = [ Above Average ]

3. Target safety score = [ Low ]

4. Target correlation score = [ Below Average ]

5. Pathway enrichment analysis score = [ Same ]

6. The final score of the machine learning model = [0.93], ranked 4 out of 24753 candidates

7. In the top 1000 dual-target rankings with [CD274], [CTLA4] ranks 1 out of 96 candidates

8. In the top 1000 dual-target rankings with [CTLA4], [CD274] ranks 1 out of 69 candidates
